# Determinants of cognitive function in childhood: A cohort study in a middle income context

**DOI:** 10.1186/1471-2458-8-202

**Published:** 2008-06-06

**Authors:** Darci N Santos, Ana Marlúcia O Assis, Ana Cecília S Bastos, Letícia M Santos, Carlos Antonio ST Santos, Agostino Strina, Matildes S Prado, Naomar M Almeida-Filho, Laura C Rodrigues, Mauricio L Barreto

**Affiliations:** 1 Department of Collective Health, Institute of Collective Health, Federal University of Bahia, Salvador, Brazil; 2 Department of of Nutritional Science, School of Nutrition, Federal University of Bahia, Salvador, Brazil; 3 Department of Psychology, School of Philosophy and Human Sciences, Federal University of Bahia, Salvador, Brazil; 4 Department of Infectious and Tropical Diseases – London School of Hygiene & Tropical Medicine, London, UK

## Abstract

**Background:**

There is evidence that poverty, health and nutrition affect children's cognitive development. This study aimed to examine the relative contributions of both proximal and distal risk factors on child cognitive development, by breaking down the possible causal pathways through which poverty affects cognition.

**Methods:**

This cohort study collected data on family socioeconomic status, household and neighbourhood environmental conditions, child health and nutritional status, psychosocial stimulation and nursery school attendance. The effect of these on Wechsler Pre-School and Primary Scale of Intelligence scores at five years of age was investigated using a multivariable hierarchical analysis, guided by the proposed conceptual framework.

**Results:**

Unfavourable socioeconomic conditions, poorly educated mother, absent father, poor sanitary conditions at home and in the neighbourhood and low birth weight were negatively associated with cognitive performance at five years of age, while strong positive associations were found with high levels of domestic stimulation and nursery school attendance.

**Conclusion:**

Children's cognitive development in urban contexts in developing countries could be substantially increased by interventions promoting early psychosocial stimulation and preschool experience, together with efforts to prevent low birth weight and promote adequate nutritional status.

## Background

The effects of poverty on child health and development are cumulative and also affect the multiple contexts of childrens' lives including factors from both proximal and distal levels [1]. Children who are persistently poor when compared with their non-poor peers, show large deficits in cognitive and social-emotional development. The long-term poor score significantly lower on cognitive achievement tests than do children who are not poor [2]. Links between socioeconomic status (SES) and cognitive performance apply in many societies, and a cross-cultural review has found that socioeconomic indicators are strongly related to cognitive development from infancy to middle childhood [3].

Low socioeconomic status can be understood as a distal risk factor that acts by mediating risk mechanisms for families with a direct influence on child development [4]. The connection between socioeconomic status, stimulating experiences and children's cognitive functioning is well established [5-7]. Stimulation provides both direct and indirect learning opportunities and servies as a motivational base for continued learning [8]. Income, education and occupation have been found to be positively associated with better parenting, which in turn affects school achievement via skill-building activities and school behaviour.

It has been argued that children of low socioeconomic status lack cognitively stimulating materials and experiences, which limits their cognitive growth and reduces their chances of benefiting from school [9,10]. Stimulating materials and experiences mediate the relationship between socioeconomic status or family income and children's intellectual and academic achievement, from infancy to adolescence [11].

However few studies have examined the relationship between poverty and the contexts of interactions in the household. Apart from the direct influence of income on material resources, economic limitations make it more difficult for poor parents to provide intellectually stimulating facilities such as toys, books, and day care, which contribute to children's development. In addition, stressed parents can be less responsive to the child and more likely to punish their children more severely. Poverty can affect many different aspects of children's lives, and its effects are examined through the 6 dimensions of the HOME inventory [12].

Home environment and parent-child interaction, as measured by the Home scale, explain some of the differences between poor and non-poor children's cognitive outcomes [13]. The physical quality of the home environment has also been linked to children's intellectual and social wellbeing [14,15]. Latin America studies have found an association between measurements of the quality of children's environments and their intellectual performance [16,17].

In addition to family-level influences such as differences in parenting style, the neighbourhood has been shown to exert an effect on chidren's psychological development. It has been shown that living in areas with high proportion of people with a good income positively affects the IQ of five year-olds [13]. It is important to consider community-level socioeconomic status because the neighbourhood in which children live has been associated with children health, achievement and behavioural outcomes, even after controlling for individual-level income and education [18].

Socioeconomic status has an impact not only on cognitive development but also on health. Children from families of low socioeconomic status are more likely to experience growth retardation, be born prematurely, and present low birth weight [19]. Low socioeconomic status is associated with iron deficiency [20] and stunting [6].

Therefore when estimating the independent contribution of children health (parasitic infection, malnutrition, and diarrhea) on cognitive score, it is necessary to consider the contribution of proximal factors such as the quality of the psychosocial stimulation.

One of the main limitations of research on socioeconomic status is the failure to consider simultaneously correlated mediating factors when studying how one particular factor operates to influence a specific developmental outcome: Children who experience inadequate nutrition are also more likely to be exposed to environmental hazards and to receive inadequate parenting. To establish a link between socioeconomic status and cognitive function via poor nutrition, it may be critical to consider also access to cognitively stimulating materials and experiences and investigating the impact of inadequate nutrition on cognitive development [21].

Brazilian studies on cognition have focused mainly on children's developmental problems relating to diarrhoea, prematurity and low birth weight. For instance, cognitive performance at pre-school age was longitudinally assessed for 79 very low birth weight premature children, with a mean score of 75.6(± 11.9) and ranging from 48 to 111 points, as measured on the WPPSI-R [22]. Among preschool children with birth weight less than 2500 g, the mean cognitive performance on the Griffiths Scale was 93,7 although it was positively correlated with birth weight [23]. Another study assessed cognitive function among 26 six to nine-year-old children in a northeast Brazilian shantytown who had undergone full surveillance for diarrhoea over their first two years of life, and found a negative association between diarrhoea burden and three measurements of cognitive function [24]. However one question to be raised in relation to these and several other findings of associations between poor cognitive function and early health and nutritional status is whether the main association was adequately controlled for confounders.

The question concerning the possible association between early health and nutritional factors and cognition has been recently addressed by a well designed Peruvian study that used a complex set of variables and controlled for several potential confounders. It identified an association between malnutrition and *G. lamblia *infection on cognitive performance [25]. However, even this study did not include any variable related with psychosocial stimulation, which is a relevant factor related with cognitive performance, [26] and consequently an important potential confounder of the role of poor nutrition and infection on cognitive performance. Inadequate psychosocial stimulation, undernutrition and infections are more common among poor children, and psychosocial stimulation, along with the physical quality of the home environment, is recognized as an intermediate level in the determination process, mediating the effect of socioeconomic status on children's cognitive development [13]. It is therefore logical to argue that failure to control for psychosocial stimulation could lead to an overestimation of the impact of nutritional deficit and infections on the cognitive deficit.

As it is not yet fully known how the various components of socioeconomic status interact with other aspects of family, neighbourhood context and children's individual health to affect cognitive performance, we put forward the aim of examining how socioeconomic status operates through multiple factors simultaneously to affect cognitive performance. We hypothesised that family material resources and status would be important determinants of cognitive outcomes among poor urban Brazilian children and that the effects of poverty would be partially mediated by poor environmental quality (both in the household and in the neighbourhood) and lower psychosocial stimulation and these effects on cognitive score would not be fully dependent on children's health and nutritional status. In order to examine this hypothesis, and attempting to overcome the short-comings of previous research a rich set of data at the individual, household and neighborhood levels would be required.

**Figure 1:**
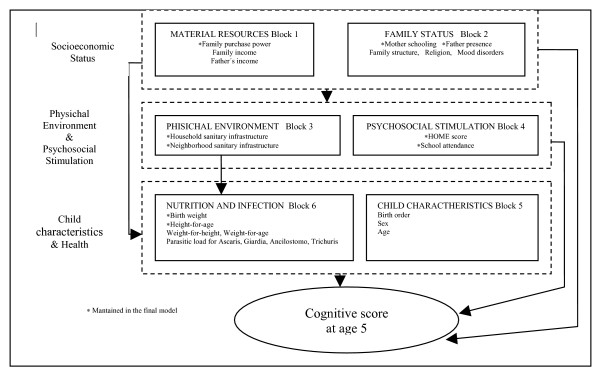
Conceptual framework.

The aim of this study was to examine the impact of poverty on cognitive scores at five years of age, by breaking down the possible causal pathways through which poverty affects cognition. This enabled us to consider the mediating effect of psychosocial stimulation and a wide range of household environment, neighborhood environment, child health and nutritional status variables. The framework in Figure 1 shows the proposed pathway for testing the ways in which the most distal socio-economic factors and a set of mediators would affect cognitive scores in later childhood.

## Methods

### Study setting

The study was conducted in Salvador, northeastern Brazil, with a population of approximately 2.5 million people, of which about 300,000 children under five years [27]. Salvador has a high degree of social inequality, but over 95% of the households are connected with piped water and a large sanitation programme is expanding the sewage system from 25% in the middle 1990s to over 60% [28].

### Study design

A longitudinal study of cognitive performance of pre-school children was conducted between January 1999 and December 2001. The cohort in the cognitive study was part of a cohort followed from 1997 for a diarrhoea and sanitation study. Children who were younger than 42 months during the first six months of 1999 were eligible to participate in the cognitive study sub-sample. Their mean age was 31.7 months (SD 5.5), ranging from 14.2 to 42.1 months

### Enrollment

The selection process of study areas [29] and population for the original cohort [30] has been described elsewhere. Briefly, for the diarrhoea study a cohort of 1,156 children under 3 years at baseline was randomly selected from 30 "sentinel areas" to represent the range of socio-economic and environmental conditions in Salvador. This cohort was created to study diarrhoea morbidity, intestinal parasitic infection and growth as part of a research effort aimed at evaluating the impact of a city-wide environmental sanitation programme [31]. To meet the original purpose of the study, the follow-up terminated in April 1999.

**Figure 2:**
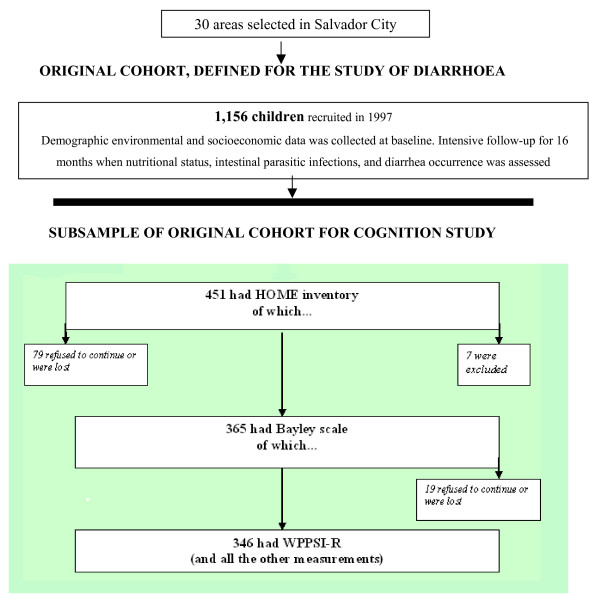
Flow chart demonstrating selection of children from the initial cohort to the present study.

The families of 365 children of the original cohort, who were under 42 months before June 1999, agreed to participate in this extended follow up, completing data on domestic psycho-social stimulation and cognitive function (Figure 2). The cognitive performance at five years old, measured by the Wechsler Pre-School and Primary Scale of Intelligence Revised (WPPSI-R)-[32], was the main outcome, and it was evaluated in 2001, in 346 children who completed the full assessment (Figure 2). No significant difference was found, as for several confounding variables, between the children who remained in the study and the 19 ones who were lost.

The data analyzed here were either retrieved from the previous study, or collected at two points, in 1999 and in 2001. In the previous study a team of field workers who had completed secondary education visited households twice weekly from December1997 to April 1999, collecting data on birth weight, parasitic infection, diarrhea, nutritional status mother's schooling and sanitary conditions. The measurement of psychosocial stimulation in 1999 was also conducted in the household by a team of four psychology interns and a licensed psychologist working as a supervisor. The measurement of cognitive performance in 2001 was conducted by a second team of four psychology interns and a licensed psychologist working as a supervisor, who also collected data on pre-school attendance and family purchasing power. Two junior psychiatrists assessed lifetime mood disorders in parents/carers. No information from children's medical records was used.

### Nutritional status

Child's weight and height/length were measured at baseline with Filizola microelectronic scales and portable stadiometer, respectively-[33].

### Morbidity

In the original cohort, children were followed-up for a maximum 16 months period for diarrhoea morbidity, being surveyed twice-weekly for diarrhoea occurrence on the 3–4 previous days. A day with diarrhoea was defined by the occurrence of three or more loose or liquid motions [30]. Longitudinal prevalence – that is, the fraction of days of follow-up with diarrhea, which has been shown to be more closely associated than incidence with long-term health effects such as weight gain and mortality [34] – was used as the measure of diarrhea burden. Stool samples were collected once between May and September 1998 and examined for helminths and protozoa. The modified Kato method [35] and sedimentation technique were employed.

### Psychiatric evaluation of parents

Carers' mood disorder was assessed with the Composite International Diagnostic Interview (CIDI) [36] in 2001.

### Stimulation

The Home Observation for Measurement of the Environment Inventory (HOME) [37], is an instrument-based questionnaire that provides a summary value which estimates the quality of psychosocial stimulation a child receives at home. This is based on characteristics of the home environment and interactions between mother and child. It has been translated into Portuguese and used in previous studies-[38]. In our study the inter-rater reliability was assessed in a sub-sample of 56 children with a very high intraclass correlation coefficient for the full scale. An adequate range may vary between 29 and 32 points.

### Cognitive performance

Cognitive functioning at age 5 was assessed using the WPPSI-R instrument. The full scale intelligence quotient has a reliability coefficient of 0.96, and a test-retest stability coefficient of 0.91 [32]. It has been translated into Portuguese and previously applied in Brazil [39]. We assessed inter-observer reliability to verify agreement in the application of the scale. Intraclass correlation coefficients were very high: 0.97 (full scale IQ), 0.95 (verbal IQ) and 0.98 (performance IQ). The range on this scale may vary from 50 to 150 points, and a range of 85 to 115 with an average performance of 100 points is considered adequate.

Both rounds of cognitive measurements (1999 and 2001) were conducted by licensed psychologists. Psychiatrists trained in the Brazilian version applied the CIDI [36] to the carer at home.

### Conceptual model: levels, blocks and variables

**Figure 3:**
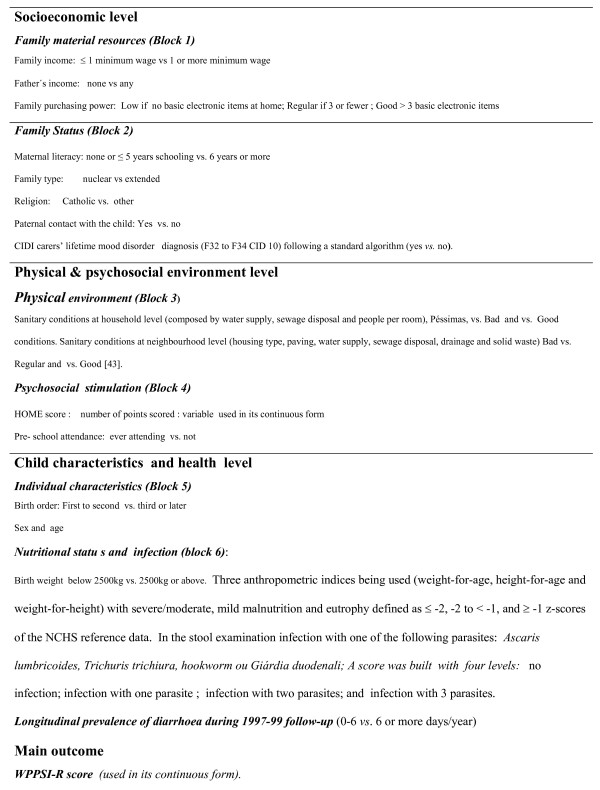
Variables definitions.

Variables were grouped in levels and blocks defined according to the proposed causal mechanism expressed by the conceptual framework (Figure 1) and defined in Figure 3. Three levels were defined, from distal to proximal: socio-economic status, physical and psychosocial environment, and child characteristics and health. The levels included a number of blocks, and each block a certain number of variables. The first level includes two blocks, one referred to material resources (family purchase power, family income and father's income) and the other one to family status (mother's literacy, father's presence, family type, religion.) determinants. Two, also, are the blocks at the second level, one including determinants of the physical environment quality (household sanitary infrastructure, neighborhood sanitary infrastructure) and the other one with the variables of psychosocial stimulation (HOME score and pre-school attendance). The third level (proposed to mediate a part of the effect of the physical environment) includes two blocks of determinants: those related to the child's charactheristics (birth order, sex and age), and those related to nutritional status (birth weight, height & weight for age, weight for height) diarrhea and parasitic load (for Ascaris, Giardia, Ancilostomo, Trichuris). The dependent variable was cognitive score at age 5, measured by the WPPSI-R instrument.

### Statistical analysis

Three steps were used for analyzing the relationship between the complexity of determinants organized on three levels and the WPPSI-R cognitive score at age five as the dependent variable: first, a one-way analysis of variance (ANOVA) with each explanatory variable; second, a multivariable ANOVA analysis within each block assessing the independent role of variables from each block; (the variables found significant in this last analysis were included in the multivariate ANOVA model); finally, a multivariate ANOVA model, in which blocks of variables from more distal to more proximal levels are added in sequence following a hierarchical approach defined by the conceptual framework (Figure 1). An effect-decomposition strategy was applied [41,42] to fit seven linear regression models (A, B, C, D, E, F, G) by including step-by-step blocks of potential risk factors. The 10% level of significance was used as the criterion for the variable inclusion in the blocks of the conceptual framework. By comparing the risk estimates obtained with the different models, we were able to examine the pathways by which the risk factors act on cognitive score. For a variable to remain in the fitted hierarchical models, a p-value less than 5% was necessary.

Significance of the contribution of each variable was assessed with the F-test after adjustment for the effects of variables already included. Regression coefficients were used to estimate the effect of each variable in the WPPSI-R cognitive scores according to levels for categorical variables, and a one-unit increase in continuous variables. The multiple coefficient of determination (R^2^) was used to measure the percentage of variance in the cognitive score explained by the independent variables for each step in the analysis. The assumptions of normal distribution and equal variance around the mean required by multiple linear regression were satisfied. The models seemed a good representation of the data, according to residual analysis. We tested product terms for the socioeconomic and environmental levels (Model D), as well as for the socioeconomic, environmental and child-health levels (Model G). The Wald test was used to assess the statistical significance of the product terms. We used SPSS (version 10.1) for all analyses.

### Ethical issues

Ethical committee approval was granted by the Research Committee of the University Hospital Professor Edgard Santos, Federal University of Bahia. Informed consent was signed by the parent or main career of all participating children. During the study children who presented health problems were treated or advised to seek medical care.

## Results

### Description of the sample

At the final examination, children had a mean age of 59 months (SD 5.4), and 55% were boys. 80% reported previous or current pre-school attendance. A prevalence of 23% lifetime mood disorders for carers was found. Around 22% of the households had only 3 goods: a refrigerator, radio and TV; 37% of families had income equal to less than a local minimum wage. 55% were nuclear families, with 62% Catholic. 28% of mothers had 5 years schooling or less; 16% of children had no contact with the father. 40% of homes and 30% of neighbourhoods had good sanitary conditions. 15% of the children were born weighing 2500 g or less and 70% were the first or second born. The mean score for domestic stimulation was 27.1 (SD 5.5). The mean WPPSI-R cognitive score was 82.6 points (SD 13.7). Severe/moderate malnutrition (equal to or below -2 z-scores), was 20.8%, 25.1% and 17.9.0% for height-for-age, weight-for-height, and weight-for-age, respectively (Table 1). A prevalence of diarrhoea of 6 days/year or greater was found for 15.3% of the children and only 18,5% of the children were free of any parasitic infection.

**Table 1:** Means for WPPSI-R test scores according to all independent variables, defined in their risk factor groups

	**N (%)**	**Mean (SD)**
**Socioeconomic Level**		
**Family material resources (Block 1)**		
Family income		
≤ 1 minimum monthly salary]	126 (36.5)	77.60 (11.39) *
More than 1 minimum monthly salary	219 (63.5)	85.54 (14.20)
Father's income		
none	88 (25.4)	78.66 (12.19)*
any	258 (74.6)	83.98 (14.01)
Family purchasing power		
Low	76 (21.9)	75,97 (10.64)
Regular	193 (55.8)	81,49 (11.58) *
Good	77 (22.3)	92,05 (16.43)

**Family Status (Block 2)**		
Maternal literacy		
none or ≤ 5 years of schooling	98 (28.3)	76.64 (11.39) *
> 5 years of schooling	248 (71.7)	85.00 (13.90)
Family type		
non-nuclear	154 (44.5)	80.73 (13.44)*
Nuclear family	192 (55.5)	84.15 (13.84)
Religion		
Catholic	214 (61.8)	82.00 (13.96)
other beliefs	132 (38.2)	83.65 (13.38)
Father's contact with the child		
None	53 (15.7)	77.28 (13.19)*
Some	285 (84.3)	83.79 (13.68)
CIDI: mood disorders among carers		
yes	80 (23.6)	83.48 (14.01)
no	259 (76.4)	82.29 (13.76)

**Physical & Psychosocial Environment Level**		
**Physichal environment (Block 3)**		
Household sanitary conditions		
Very poor	69 (19.9)	76.01 (11.16)
Poor	136 (39.3)	81.78 (11.82) *
Good	141(40.8)	86.69 (15.21)
Neighbourhood sanitary conditions		
Bad	71 (20.5)	76.14 (12.16)
Regular	174 (50.3)	81.11 (12.02)*
Good	101 (29.2)	89.80(14.58)
**Psychosocial stimulation (Block 4)**		
Below the mean	195 (56.53)	78.51 (11.26) *
Above the mean	150 (43.47)	87.73 (14.64)
Child attendance at nursery school		
Never attended	66 (19.07)	72.71 (10.70) *
Attended at some time	280 (80.93)	84.97 (13.35)

**Child Characteristics And Health Level**		
**Child characteristics (Block 5)**		
Birth order		
Third or subsequent	103 (29.8)	79.06 (13.00)*
First or second	243 (70.2)	84.41 (13.66)
Sex		
Female	157 (45.4)	82.87 (12.91)
Male	189 (54.6)	82.43 (14.43)
Age		
Up to 59.68 months	186 (53.8)	83.99 (13.91)*
More than 59.68 months	160 (46.2)	81.04 (13.42)
**Nutrition and infection (Block 6)**		
Birth weight		
≤ 2500 g	50 (14.5)	77.27 (14.12) *
More than 2500 g	296 (85.5)	83.78 (13.39)
Height for age		
≤ -2 Z scores	72 (20.8)	77.65 (15.19) *
> -2 Z scores	274 (79.2)	83.94 (13.06)
Weight for age		
≤ -2 Z scores	87 (25.1)	78.49 (14.11)*
> -2 Z scores	259 (74.9)	84.02 (13.36)
Weight for height		
≤ -2 Z scores	62 (17.9)	78.95 (14.09)
> -2 Z scores	284 (82.1)	83.43 (13.56)*
Diarrhoea prevalence:		
More than 6 days/year	53 (15.3)	79.13 (15.27)
Below	293 (84.7)	83.26 (13.39)
Number of intestinal parasitic infection:		
None	64 (18.5)	87.31 (17.685)*
One parasite	206 (59.5)	83.10 (12.22)
Two	56 (16.2)	79.41 (12.34)
Three or more	20 (5.8)	71.80 (10.57)

* statistically significant difference at 5% level

Table 2 shows the results of the first and second of the three-step analysis. β estimates the increase in cognitive score for each unit increase in the variable; R^2 ^estimates the proportion of the variation in cognitive scores explained by the variable (in the univariable analysis) or by the group of variables (in the multivariable analysis), or by each model in the hierarchical analysis. In Step 1 (univariable analysis) most variables in all blocks were statistically associated with the WPPSI-R cognitive score. Associations with family purchasing power, household sanitary conditions, HOME score and pre-school attendance are noticeable, each one explaining (R^2 ^value) 12% or more of the variation in cognitive score at age 5. The contribution of the variables in the nutrition and infection block to variation in cognitive score at age 5 were generally modest; for *intestinal parasites *infection the association was statistically significant, at univariate and multivariable analysis, but the significance did not remain in the hierarchical analysis.

**Table 2:** Univariable and multivariable analysis of determining factors grouped by blocks and levels with WPPSI-R cognitive scores.

**Blocks And Variables**	**Univariable analysis**		**Multivariable analysis (by block)**	
		
	**R^2 ^adjusted (%)**	**βu (95% CI)**	**R^2 ^adjusted (%)**	**βk (95% CI)**
**Level 1 Socioeconomic status**				
**Material Resources (Block 1)**			**17.0**	
Family purchasing power	15.5			
Low				
Regular		5.51 (2.15; 8.88)		4.4 (0.9; 7.8)
Good		16.0 (12.0; 20.0)*		13.7 (9.3; 18.0)*
Family income	7.7			
≤ 1 minimum wage				
More than 1 minimum wage		7.9 (5.0; 10.8)*		4.0 (1.0; 7.0)
Father without income	2.6			
None				
Any		5.3 (2.0; 8.6)		
**Family Status (Block 2)**			**9.7**	
Mother's schooling	7.5			
none or ≤ 5 years				
> 5 years		8.3 (5.2; 11.4)*		8.2 (5.1; 11.3)*
Father-child contact	3.0			
None				
Some		6.5 (2.5; 10.5)*		5.9 (2.1; 9.8)*
Family structure	1.5			
non-nuclear				
Nuclear family		03.4 (0.5; 6.3)*		
Religion	0.3			
Catholic				
other beliefs		-1.6 (-4.6; 1.3)		
CIDI mood disorders among carers (2001)**	0.0	-1.9 (-3.6; 3.2)		

**Level 2 Physical Environment and Psychosocial Stimulation**				
**Sanitary conditions (Block 3)**				
Household	12.7		**15.3**	
Very poor				
Poor		4.9 (1.4; 8.5)		3.7 (0.2; 7.3)
Good		13.6 (9.7; 17.5)*		11.2 (7.1; 15.3)*
Neighbourhood	7.8			
Bad				
Regular		5.8 (1.9;9.6)		4.6 (0.8; 8.3)
Good		10.7 (6.9; 14.5)*		7.0 (3.1; 10.9)*
**Psychosocial Stimulation (Block 4)**				
HOME score (1999)	16.3	0.9 (0.7; 1.2)*	**23.7**	0.8 (0.6; 1.0)*
Nursery school attendance	12.3			
Never attended				
Attended at some time		12.2 (8.7; 15.7)*		9.8 (6.5; 13.0)*

**Level 3 Child characteristics and health**				
**Child's Characteristics (Block 5)**				
Birth order	2.9		**2.3**	
Third or subsequent				
First or second		5.0 (1.9; 8.2)*		5.0 (1.9; 8.2)*
Sex	00			
Female				
Male		-0.4 (-3.3; 2.4)		
Age	1.1			
Up to 59.68 months				
More than 59.68 months		-2.9 (-5.9; -0.04)		
**Nutrition And Infection (Block 6)**			**9.8**	
Birth weight	2.8			
≤ 2500 g				
More than 2500 g		6.5 (2.4; 10.6)*		4.8 (0.8; 8.8)*
Height-for-age (1998)	3.9			
≤ -2 Z scores				
> -2 Z scores		5.3 (2.5; 8.2)*		5.3 (1.8; 8.8)*
Weight-for-height (1998)	1.5			
≤ -2 Z scores				
> -2 Z scores		3.6 (0.5; 6.6)*		
Weight-for-age (1998)	2.8			
≤ -2 Z scores				
> -2 Z scores		4.2 (1.6; 6.9)*		
Diarrhoea prevalence (1997–1999)	1.2			
more than 6 days/year				
0–6 days/year		4.1 (0.1; 8.1)*		
Number of intestinal parasitic infection	6.2	-4.6 (-6.5; -2.7)*		-4.0 (-5.8; -2.2)*

** Measurement date

βk = β value for multivariable analysis within the block

*p < 0.05

Results of the multivariable analysis within each block showed that material resources and family status explained respectively 17.6% and 9.7% of the variation in cognitive score. Higher proportions of variation were explained by each block at the environmental level. The blocks in child health level explained around 6.0% of the variation in late cognitive score.

In Table 3, the effect from three levels and their six blocks were decomposed into seven regression models following the sequence proposed in the conceptual framework (Figure 1):

**Table 3:** Results from multivariable hierarchical analysis of determining factors grouped by levels with WPPSI-R cognitive scores.

**Variables**	**Model A (Blocks 1 and 2)**		**Model B (Blocks 1, 2 and 3)**		**Model C (Blocks 1, 2 and 4)**		**Model D (Blocks 1, 2, 3 and 4)**			**Model E (Blocks 1, 2, 3, 4 and 5)**		**Model F (Blocks 1, 2,3,4 and 6)**		**Model G (Blocks 1, 2, 3, 4, 5 and 6)**		
							
	**R**^2^	**β(95% CI)**	**R**^2^	**β(95% CI)**	**R**^2^	**β(95% CI)**	**R**^2^	**β(95% CI)**	Change β **	**R**^2^	**β(95% CI)**	**R**^2^	**β(95% CI)**	**R**^2^	**β(95% CI)**	Change β***
**Level 1 Socioeconomic status**																

**Socioeconomics**	**20.2**		**26.3**		**30.1**		**34.4**			**39.8**		**35.3**		**40.3**		
**Family purchasing power**									-60.9							-25.0
Low																
Regular		3.9 (0.5; 7.3)**		2.7 (-0.5; 6.0)**		2.5 (-0.6; 5.6)**		1.5 (-1.6; 4.6)**			0.4 (-2.6; 3.5)**		1.3 (-1.7; 4.4)**		0.3 (-2.7; 3.3)**	
Good		13.3 (9.3; 17.4)		9.5 (5.2; 13.7)		8.1 (4.1; 12.1)		5.2 (1.1; 9.3)			5.1 (1.1; 9.1)		5.0 (0.9; 9.1)		4.9 (0.9; 8.9)	
**Family characteristics**																
**Mother's schooling**																
*None or *≤ *5 years*																
> *5 years*		5.4 (2.3; 8.5)		5.7 (2.8; 8.6)		3.9 (1.1; 6.7)		3.9 (1.1; 6.7)	-27.7		3.6 (0.9; 6.3)		3.7 (1.0; 6.5)		3.5 (0.8; 6.1)	-66.0
**Father-child contact**																
None																
Some		4.8 (1.1; 8.5)		4.3 (0.7; 7.8)		4.1 (0.7;7.5)		3.5 (0.2; 6.8)	-27.0		3.9 (0.6; 7.1)		3.6 (0.3; 6.9)		3.9 (0.6; 7.1)	-8.5

**Level 2 Physical environment and Psychosocial stimulation**																

**Sanitary conditions**																
**Household**																
Very Poor																
Poor																
Good				5.9 (1.7; 10.1)				4.7 (0.7; 8.7)			4.7 (0.9; 8.5)		4.2 (0.2; 8.1)		4.3 (0.5; 8.2)	-40.0
**Neighbourhood**				0.4 (-0.8; 3.0)**				0.7 (-2.5; 4.0)**			1.2 (-1.9; 4.4)**		0.2 (-3.0; 3.5)**		0.9 (-2.2; 4.1)**	
Bad																
Regular																
*Good*				6.2 (2.5; 9.9)				5.8 (2.4; 9.3)			6.1 (2.8; 9.5)		6.0 (2.6; 9.4)		6.2 (2.9; 9.6)	-22.4
**Psychosocial stimulation**				5.0 (0.0; 1.5)**				4.5 (1.1; 7.8)**			5.3 (2.1; 8.5)**		4.5 (1.2; 7.8)**		5.3 (2.1; 8.5)**	
**HOME **score (1999)																
**Nursery school attendance**						0.6 (0.3; 0.8)		0.5 (0.3; 0.7)			0.5 (0.3; 0.8)		0.5 (0.3; 0.7)		0.5 (0.3; 0.7)	00.0
Never attended						7.8 (4.5; 11.1)		7.3 (4.1; 10.5)			8.4 (5.2; 11.5)		6.9 (3.7; 10.1)		8.0 (4.9; 11.2)	-12.0
Attended at some time																

**Level 3 Child characteristics and Health**																

**Nutrition and Infection**																
**Birth weigh**																
≤ 2500 g																
More than 2500 g																
**Height-for-age**											6.6 (3.3; 9.9)		3.5 (0.5; 6.5)		6.0 (2.7; 9.4)	
≤ -2 Z scores																
> -2 Z scores															2.9 (-0.2; 5.8)	

Change β** = (βD-βA)/βA (%); Change β*** = (βH-βD)/βD (%)

*p < 0.05

**Model A **(only blocks 1 and 2) sought to estimate the overall effect of socioeconomic status level. This alone explained 20.2% of the variation in cognitive score, with beta values of 13.3 points difference in the score for children living in a family with improved purchase power, 5.4 points if the mother had above five years schooling, and 4.8 points if the father was present.

In **Model B **(included in addition significant variables from block 3) sought to estimate the effect of socioeconomic level not mediated through sanitary conditions. The variation explained increased to 26.3% and the beta values for purchasing power, mother's schooling and father-child contact decreased to 9.5; 5.7; and 4.3 respectively, suggesting that this loss is accounted for by the effect of socioeconomic status this is mediated by physical environment conditions. **Model C**, (psychosocial stimulation variables (block 4) included) **C **explained 30.1% of the variation and beta values for purchasing power, mother's schooling and father-child contact, decreasing even more.

**Model D **(variables of blocks 1, 2, 3 and 4 combined) sought to estimate the effects of socio-economic level not mediated through physical environment and psychosocial stimulation. Comparing effect estimators through the previous three models, beta values for socioeconomic status decreased after adjusting for blocks 3 and 4 at the physical and psychosocial environmental level. A 13.3 points difference in the cognitive score for children living with parents with better purchasing power in **Model A**, decreased to 9.5 points after adjusting for sanitary conditions in block 3 (**Model B**) and to 8.1 points after adjusting for block 4, psychosocial stimulation (**Model C**). After adjusting for blocks 3 and 4 combined the differences came to 5.2 points for purchasing power, 3.9 for mother's schooling and 3.5 points for contact with the father (**Model D**). This Model estimated the effects of social-economic level not mediated through the intermediate environmental level, as well as the overall effect of variables for sanitary conditions (block 3) and psychosocial stimulation (block 4) on cognitive score. The magnitude of the effect of the environment (level 2 **Model D**), did not change substantially after adjusting for the variable of block 5 (child at birth), as presented in **Model E**. Apart from an increase in the effect of pre-school experience from 7.3 to 8.0 points on cognitive score, the beta values for HOME score, household and neighbourhood conditions remained substantially the same.

**Model E **(variables of blocks 1, 2, 3, 4, and 5) sought to estimate the effect of the physical and psychosocial environment level not mediated by the health of the child at birth (block 5), and the overall effect of variables in this block. A child born with normal birth weight had 6.7 points advantage in the cognitive score.

**Model F **(variables of blocks 1, 2, 3, 4, and 6) sought to estimate the effect of physical and psychosocial environment level not mediated by nutrition and infection, and the overall effect of variables at this block. By including indications for linear growth in this Model there were very small changes in the beta estimates for environmental and stimulation variables.

Finally, **Model G**. (variables of blocks 1, 2, 3, 4, 5 and 6) sought to estimate the effect of physical and psychosocial environment levels (blocks 3 and 4), not mediated by the child health level (blocks 5 and 6), and the overall effect of the variables in the blocks in the child health level. Comparing effect estimators from **Model D level 2**, beta values for household and neighborhood sanitary conditions, HOME score and pre-schooling experience seemed very stable after adjusting for blocks 5 and 6 at the child health proximal level. A 4.7 and 5.8 points difference in the cognitive score for children living with better household and neighborhood respectively in **Model D**, corresponded to 4.4 and 6.3 points after adjusting for birth weight (**Model E**) and 4.2 and 6.0 points after adjusting for height-for-age (Block F) After adjusting for blocks 5 and 6 combined the differences came to 4.3 and 6.2 points for household and neighborhood (**Model G**). Estimates for HOME score remained the same throughout the 3 models with minimal changes for pre-school attendance.

## Discussion

We have shown that cognitive function at age five was negatively associated with poor socio-economic conditions, poor maternal education, paternal absence, inadequate sanitary conditions at home and in the neighbourhood, low birth weight, and stunting; and positively associated with high levels of domestic stimulation, and pre-school attendance. A hierarchical model constructed according to a defined conceptual framework explained 40.3% of the variation of cognitive scores at age 5. These findings are consistent with previous studies [43,44]. An effect-decomposition approach showed that the effect of poor socioeconomic conditions on cognitive performance was mediated mostly by lack of psychosocial stimulation and inadequate sanitation conditions at home and in the neighborhood. By contrast poor sanitation conditions and poor psychosocial stimulation apparently act directly on risk for inadequate cognitive performance at pre-school age, and in this population at least, does not appear to be mediated by infection or nutrition differently from previous findings. Despite the occurrence of diarrhea and intestinal parasite infections in the children studied, their burden was lower than in other populations where these associations were found.

In our study more than 5 years maternal education improved the mean WPPSI-R score by an additional 3.9 points, this being almost similar to the presence of the father with 3.5 points, increasing to 5.2 points when the family had good purchasing power. These effects were attributable to the most distal block of variables included in the socioeconomic level.

The socioeconomic effect mediated through the intermediate level shows the importance of physical and psychosocial quality of children's environment with almost 6 points difference for neighborhood environment quality and a 7.3 points increase for school attendance. It has been suggested that the community's physical structure influences health directly (through risk exposure) and indirectly (creating an environment that induces neglect of health) [44,45]. The inner household environment quality and domestic stimulation were also strong mediators in the improvement of cognitive score. Living in a house with good sanitation conditions would give an advantage of 4.7 points, and for each unit increasing in domestic stimulation there was a half point improvement for cognitive score. This is in line with knowledge concerning the role of environment on children's development [46]. In this block, pre-school attendance showed an important effect on cognitive performance, adding 7.8 points on average to cognitive scores. In older age groups, schooling has been found to be strongly associated with higher scores on cognitive development [45]. Environmental conditions remained very influential throughout the analysis as its effect was not so much modified by the child health block of factors. The value of beta for school attendance lost only 12% of its magnitude from model D to the final Model G, giving a 22% loss for neighborhood estimates and 40% loss for inner household quality and domestic stimulation.

Following the effects of children's individual health variables in the proximal level, the overall effect came to 6 points for low birth weight and 2.9 points for linear growth deficit. Low birth weight remained a strong risk factor in the final model. Other studies have already found cognitive measures increasing with birth weight [47,37]. Motor problems and low verbal intelligence coefficient at age five have been found for children with birth weight below 2000 g [48]. In our study population 12.6% had been low birth weight babies; 4.4% below 2000 g.

The socioeconomic effects mediated by the environment was only very marginally reduced with consideration of health variables which corresponded to an 8% reduction in the mean cognitive score provided by a household in good sanitary conditions, a 6% reduction for good quality neighborhood, and a 9.5% reduction for attending pre-school but no reduction at all in the domestic stimulation benefit.

Thus the effect of socioeconomic factors on cognitive score which is mediated by quality of the physical environment and psychosocial stimulation is not mediated by birth weight and linear growth. Domestic stimulation was not mediated at all by factors in the health level; neighborhood and household quality and school attendance were very little mediated by health.

There is a difficulty in using tests not designed and standardized in Brazil; however, standard procedures for training and application of the WPPSI-R instrument were followed, and agreement between examiners was adequate with intraclass correlation coefficients above 0.90. The WPPSI-R scale has not been fully validated in a Brazilian population, although it has been used in other studies in Brazil [32]. The mean of 82.6 (SD 13.7) WPPSI-R score found here is low. This however is not so far from the mean 88.9 (SD 12.5) found for Peruvian children [25]. Our mean WPPSI-R scores for those with low birth weight was 77.3 (SD 14.1); another Brazilian study found 75.0 (SD 11.9) in very low birth weight children [22]. So although results tend to be low in Latin America, they are consistent. It is possible that the WPPSI-R consistently underestimates scores in this setting, as it measures outcomes related to abilities reliant on formal education and acculturation. This would not have been a problem in our study as it did not aim to classify children according to their IQ measures, but instead to analyse how a broad-range of risk factors affects children's cognitive performance at five years old.

Our analysis was based on a previous defined conceptual model as an alternative to the more traditional analysis used in epidemiology. The simple hierarchical effect-decomposition strategy here is not without limitations as consistent estimates of direct (not mediated) effects can be obtained only when there is no confounding at the level of the intermediate variable [49]. So in order to estimate the effect of socio-economic status level not mediated by physical and psychosocial environment level, we had to assume that there were no unobserved covariates associated with the blocks for psychosocial stimulation and with risk for low cognitive score. We think this assumption holds for the environmental quality block in the same intermediate level. Our conceptual model considered a wide range of potential risk factors for cognitive score determination and grouped them in meaningful blocks through statistical analysis; it seems unlikely that other unobserved factors are associated with both, the two blocks (sanitary conditions and psychosocial stimulation) in the intermediate level, and cognitive score. Therefore, the variables in the model were previously defined according to their potential role in the studied outcome. The final model was estimated in a hierarchical way. We cannot exclude the possibility that other conceptual frameworks or other strategies of inclusion of the independent variables would change the weight of these variables regarding the outcome.

## Conclusion

We can conclude that cognitive performance, which is an important aspect of children's development, was affected by socio-economic status, which itself was mediated by psychosocial stimulation at home and pre-school, and by physical environmental conditions at home and in the neighbourhood. While children's early health indicators such as birth weight and linear growth played a role in the cognitive performance, they did not mediate the effect of socioeconomic status, physical environment and psychosocial stimulation, as conceived in the original model (figure 1). The remaining effect of the socioeconomic factors must therefore act through intermediate factors not measured in this study.

Finally, we must consider the very real limitations of an observational study and that analysis and interpretation of data following a predefined conceptual framework is a complex exercise and one in which alternative explanations are often possible. The implication is that children's cognitive development in middle income urban contexts of developing countries even when in disadvantaged socioeconomic conditions could be substantially increased by interventions promoting early psychosocial stimulation, pre-school experience and nutritional improvement. High quality childcare may provide low-income children with opportunities otherwise unavailable in their developmental trajectories [50].

## Competing interests

The authors declare that they have no competing interests.

## Authors' contributions

**DNS **designed the research methods, gathered data, managed data entry, devised the analytical framework for the study, conducted the statistical analysis and wrote the first draft and edited the final version of the manuscript.** AMOA **designed and was responsible for the nutritional survey, advised on anthropometric measures and interpretation of data, reviewed the manuscript.** ACSB **training the use of HOME, analysis and interpretation of the HOME collected data, review the manuscript.** LMS **coordinated cognitive data collection and carried out assessment and interpretation of cognitive data at both points, and reviewed the manuscript.** MSP **conducted data collection for measures on child health and socio-economics status in the original cohort, and reviewed the manuscript.** AS **designed and conducted the original cohort and adviced on data interpretation and reviewed the manuscript.** CASTS **participated in the design, statistical analysis, contributed to interpretation of the data and reviewed the manuscript. **NMA–F** participated in study design, interpretation, reviewed the manuscript.** LCR **contributed to the development of the framework, interpretation of the data, and to write and edit the manuscript.** MLB **coordinated the overall cohort study and the study design, supervised data collection and interpretation, and contributed to write and edit the manuscript. All authors read and approved the final manuscript.

## Pre-publication history

The pre-publication history for this paper can be accessed here:


